# Colorectal cancer screening knowledge, attitudes and behavioural intention among Indigenous Western Australians

**DOI:** 10.1186/1471-2458-12-528

**Published:** 2012-07-18

**Authors:** Aliki Christou, Sandra C Thompson

**Affiliations:** 1 Centre for International Health, Curtin University, GPO Box U1987, Perth, Western, Australia; 2 Combined Universities Centre for Rural Health, University of Western Australia, P.O. Box 109, Geraldton, Western, Australia

## Abstract

**Background:**

Indigenous Australians are significantly less likely to participate in colorectal cancer (CRC) screening compared to non-Indigenous people. This study aimed to identify important factors influencing the decision to undertake screening using Faecal Occult Blood Testing (FOBT) among Indigenous Australians. Very little evidence exists to guide interventions and programmatic approaches for facilitating screening uptake in this population in order to reduce the disparity in colorectal cancer outcomes.

**Methods:**

Interviewer-administered surveys were carried out with a convenience sample (n = 93) of Indigenous Western Australians between November 2009-March 2010 to assess knowledge, awareness, attitudes and behavioural intent in regard to CRC and CRC screening.

**Results:**

Awareness and knowledge of CRC and screening were low, although both were significantly associated with exposure to media advertising (p = 0.008; *p* < 0.0001). Nearly two-thirds (63%; 58/92) of respondents reported intending to participate in screening, while a greater proportion (84%; 77/92) said they would participate on a doctor’s recommendation. Multivariate analysis with logistic regression demonstrated that independent predictors of screening intention were, greater perceived self-efficacy (OR = 19.8, 95% CI = 5.5-71.8), a history of cancer screening participation (OR = 6.8, 95% CI = 2.0-23.3) and being aged 45 years or more (OR = 4.5, 95% CI = 1.2-16.5). A higher CRC knowledge score (medium vs. low: OR = 9.9, 95% CI = 2.4-41.3; high vs. low: 13.6, 95% CI = 3.4-54.0) and being married or in a de-facto relationship (OR = 6.9, 95% CI = 2.1-22.5) were also identified as predictors of intention to screen with FOBT.

**Conclusions:**

Improving CRC related knowledge and confidence to carry out the FOBT self-screening test through education and greater promotion of screening has the potential to enhance Indigenous participation in CRC screening. These findings should guide the development of interventions to encourage screening uptake and reduce bowel cancer related deaths among Indigenous Australians.

## Background

Indigenous Australians are at greater risk of death following a bowel cancer diagnosis compared to their non-Indigenous counterparts due to higher rates of late stage diagnoses of the disease [1]. Many of these deaths are easily preventable through bowel cancer screening (also known as colorectal cancer (CRC) screening) which can detect bowel cancer in its early stages when the likelihood of successful treatment is high [2,3]. In Australia, a National Bowel Cancer Screening Program (NBCSP) utilising Faecal Occult Blood Testing (FOBT) commenced in 2006 providing free screening to all adults aged 50, 55 and 65 years. Further details and a brief history of the Australian program are described in an earlier review [4] and summarised in Table 1.

**Table 1:** Australia’s National Bowel Cancer Screening Program (NBCSP)

***History and implementation***
Australia is one of a few countries implementing a formal, government-funded, population-based CRC screening program. Others include the UK, Canada, France, Italy and Finland and Japan [5]. Following a pilot program in 2002–2004, the first phase of Australia’s National Bowel Cancer Screening program was rolled out in 2006 with free screening using FOBT offered to individuals turning 55 and 65 years of age. The second phase of the program began in July 2008 and expanded eligibility to all people turning 50, 55 or 65 years. The program targets only specific age groups due to limited funding and to ensure health services can cope with increased service demands [6]. However there are doubts about the evidence-base upon which these decisions have been made [7]. Eligible participants are identified through Medicare (Australia’s national health insurance scheme) enrolment and sent an invitation and FOBT screening kit to their home. After taking two consecutive samples of stool, individuals are required to return the screening kit by post to the laboratory using a reply paid envelope provided [8]. Participants and their nominated doctor are then notified of their result by mail recommendations made for follow-up if necessary. A bowel cancer screening register was established within Medicare to assist in follow-up of positive tests not undergoing colonoscopies, re-screening of participants, and to aid data collection, monitoring and evaluation [6]. The data obtained are collected via forms filled in by the individual, their GP, colonoscopist and others. Completion of forms is not compulsory; hence a large amount of information is incomplete or missing [9].
***The screening test***
The faecal occult blood test (FOBT) is a simple non-invasive self-screening test that detects small amounts of blood in the bowel motion. The FOBT is the only screening test for CRC where evidence from randomised control trials (RCT) has demonstrated a reduction in mortality, although the trials used the guaiac FOBT (gFOBT) as opposed to immunochemical FOBTs (iFOBT), the test used in Australia. A recently completed RCT using the iFOBT demonstrated increased detection and compliance, although long-term mortality benefits have yet to be demonstrated [10].
***Targeting the Indigenous population***
In 2005/2006 it was announced that Indigenous people could participate in the NBCSP from the age of 45 years due to their earlier onset of bowel cancer [11]. However, it is not clear whether FOBT kits are in fact being sent out to Indigenous people at this earlier age. Given that this would require Indigenous status to be reliably identified on Medicare enrolment records, a large proportion of at-risk Indigenous people are likely to be missing out on earlier screening.
***Challenges and controversy surrounding the Program***
The NBCSP has drawn much negative media attention which no doubt has influenced consumers’ opinions and created uncertainty regarding the legitimacy and quality of test results. It has been described as a ≤piecemeal’ program that is inadequately funded, lacking resources and effective communication strategies [12,13]. In 2009, nearly half a million testing kits were recalled and the program was temporarily suspended for six months after an unusual decline in the number of positive test results, assumed to be due to faulty kits [7]. Testing resumed in November 2009 with a new kit that was initially distributed to re-screen those who received the faulty kits and led to significant delays in rollout of the program. The new kit is recommended for use only up to 30 degrees Celsius, creating potential problems for many regions, particularly in the north of Australia.
***Funding and future of the Program***
Initial funding for the Program was only secured up until June 2011 and the future of the program was uncertain for some time [12]. Substantial lobbying and advocacy by community groups and several organisations led to a Government announcement in July 2011 to continue ongoing funding of the program. In early May 2012, the Government committed to extending the program to those turning 60 years from 2013, and those turning 70 in 2015 [14]. However, until 2017 the program will remain as it is, allowing for once-off testing of the population, even though the mortality benefit associated with screening depends participation in regular screening [15]. According to the new announcement, full implementation including biennial screening as recommended by clinical guidelines and supported by research [3,16], will be introduced in a phased process from 2017 for all Australians aged 50 to 74. Such a fully implemented program can save lives - potentially preventing up to 500 deaths per year, and would cost the Australian Government an additional $50 million per year [17].

Regular population screening with the FOBT has been shown to reduce mortality from bowel cancer [15,18,19], however participation rates of Indigenous Australians are less than half that of the general population [20]. These findings parallel CRC screening uptake rates in Indigenous and ethnic minority groups around the world, which are also disproportionately lower than mainstream populations [21-25].

Evidence from other cancer screening programs in Australia indicate that lower participation of Indigenous people results from a multitude of structural, socio-cultural and behavioural factors, including shame and fear regarding cancer, culturally insensitive services, poor literacy, distance and access barriers, and low levels of knowledge and awareness of the cancer, the screening tests available, and associated benefits of screening [26].

To successfully create targeted messages and educational resources, and to inform interventions aimed at enhancing screening participation, requires understanding of the current knowledge, attitudes and beliefs of the community of interest. To date there have not been any peer-reviewed studies investigating the knowledge and attitudes of Indigenous Australians towards bowel cancer screening, and the factors influencing the decision to engage in screening utilising FOBT.

Determinants of screening participation have been examined in the context of health behaviour theories such as the Health Belief Model [27] which provides a framework in which to study the relationship between an individual’s perceptions and beliefs about a disease, and their decision of whether to engage in a preventive health behaviour. Perceptions of personal susceptibility to, and perceived severity of the disease, belief in one’s ability to undertake a preventive action (perceived self-efficacy), the potential benefits of, and perceived barriers to undertaking this behaviour, all influence an individual’s decision to partake in a preventive action. Attitudes are also influenced by demographic and other structural or socio-psychological factors. This model purports that a cue to action must occur to trigger a behavioural change; this may result from a doctor’s recommendation, media advertisement or an intervention. Several studies have used health belief theories to understand and identify factors associated with screening compliance [28-31]. By taking a similar approach, this study aimed to elucidate factors that may be associated with CRC screening intention. We present findings from a baseline survey that was conducted as part of an intervention study to assess the current knowledge, attitudes and behavioural intentions of Indigenous Western Australians in relation to bowel cancer and screening with FOBT. This information is critical for identifying specific areas for intervention, and focus for health promotion and education, to improve uptake of CRC screening and reduce disparities in bowel cancer outcomes for Indigenous Australians.

## Methods

### Study participants and data collection

Participants were recruited from two regional and one metropolitan site in Western Australia through community and family networks of health professionals known to the researchers. Recruitment occurred between November 2009-March 2010 with assistance from Aboriginal staff employed in Aboriginal-specific health services. Eligibility criteria included self-identification as Aboriginal or Torres Strait Islander, aged 35 years or more, and the provision of written informed consent prior to participation. Questionnaires were administered at face-to-face interviews and participants received $20 to compensate for time and travel expenses. An Indigenous reference group assisted with all aspects of the study. The Western Australian Aboriginal Human Information and Ethics Committee (250 08/09) and the Curtin University Research Ethics Committee (CIH 11-2009) approved the study.

### Questionnaire

#### Survey development

The questionnaire measured respondent’s knowledge, attitudes, beliefs and behaviours related to the prevention, early detection and treatment of bowel cancer. The instrument was adapted and further developed from surveys identified in relevant literature, as no existing validated questionnaire was found that examined these elements in Indigenous Australians for any cancer screening modality. We favoured items from surveys used with minority and/or Indigenous populations and which were guided by key elements of the Health Belief Model [32,33]. Items were also adapted from the knowledge, attitudes and practices (KAP) survey conducted on the general Australian population prior to the NBCSP pilot [34]. Before implementation, the questionnaire was piloted on a small group of Aboriginal people to ensure understanding and cultural appropriateness, and modified accordingly.

#### Measures

The survey examined awareness of bowel cancer and bowel cancer screening (if they had heard about the disease or the screening test/program and to describe what they knew) and general knowledge and recognition of risk factors and signs and symptoms of bowel cancer. A knowledge score was computed for each respondent based on 18 knowledge items with scores representing the number of correctly answered items. For analysis, scores were divided into tertiles of low (0-11), medium (12-15) and high (16-18). These cut-off points were selected based on dividing our sample population in almost equal thirds so that each third contained roughly the same number of cases. As responses were distributed towards greater knowledge with very few participants having a score less than 6, it was not appropriate to divide the total score based upon being one third of the range (i.e. 0-6, 7-12, 13-18). Respondents in the bottom third were considered to have lowest knowledge, the middle third as medium knowledge, and top third as high knowledge.

Screening behaviour was determined indirectly by asking respondents about their *intention* to participate in bowel cancer screening *(‘Would you consider doing an FOBT bowel cancer screening test in the next 6-12 months if a screening kit was given or sent to you?’*), and if they would take up screening following a doctor’s recommendation. Attitude measures included perceptions of personal susceptibility to (*‘Do you believe you are at risk of getting bowel cancer?’*) and perceived severity of bowel cancer (*‘Do you think bowel cancer can be cured if found early?’*), perceived self-efficacy (‘*How confident do you feel in your ability to do the FOBT screening test?’*), perceived benefits, barriers and motivators for bowel cancer screening, fatalistic attitudes (*‘Getting cancer is like being sentenced to death’*) and perceptions of shame *(‘You would feel too ashamed if you found out you had bowel cancer’*).

Items presented in a statement format required respondents to indicate whether they agreed or disagreed, with response categories consisting of a five-point Likert scale (1 = strongly disagree, 2 = disagree, 3 = agree, 4 = strongly agree, and 5 = don’t know). Where the format of the question answer was yes, no or don’t/know, the no and don’t know categories were combined for bivariate and multivariate analyses.

Health-care seeking behaviour including past screening participation and subjective health status were assessed, as was family or personal cancer experience and exposure to media advertising on bowel cancer screening. Socio-demographic information was also collected and included self-rated English reading ability.

### Statistical methods

Questionnaire data was analysed using statistical software PASW statistics 17 (SPSS Inc., IBM Company, Chicago, Ill., USA). Descriptive statistics were carried out on all survey items. Bivariate analyses were conducted using Chi-square or Fisher’s exact test (when Chi-square assumptions were not met) to examine relationships between categorical variables. Responses were dichotomised as described above for bivariate and multivariate analyses. Binary logistic regression was used to explore the relationship between intention to screen for bowel cancer and variables showing associations at the p = 0.30 level or less in bivariate analyses to identify independent predictors of screening intent. All variables showing associations at the p = 0.30 level or less were included in the first step of the logistic regression model. Variables with ten or more missing responses were excluded, as was the item measuring awareness that bowel cancer/polyps can be asymptomatic, as this was included in the calculation of the total knowledge score. As bowel cancer knowledge score was significantly related to perceived self-efficacy, perceived susceptibility and having participated in screening in the past in bivariate analyses, all of these variables could not be included in the same logistic regression model. Thus, two logistic regressions were performed – one excluding these three variables and another including them, but excluding the knowledge variable. Marital status was almost significantly related to knowledge (p = 0.054) and initially retained in both models, however was subsequently removed from model 1 as it caused unstable odds ratios and wide confidence intervals. Thus, the first model included 12 of the 26 variables and the second included 10. A backward step-wise approach using the default ‘enter’ method in SPSS was utilised, removing variables from the model that were non-significant until only variables with a p <0.05 remained in the model.

## Results

### Socio-demographic characteristics

A total of 93 participants were recruited for this study (Table 2). Females were over-represented with less than 25% of respondents being male. The majority (47.8%) were in the 45-54 year age group, around half (52.7%) were married or living in de-facto relationships, 44% had either completed senior high school or a post-school qualification, and over half (56.7%) were unemployed or not working. Most respondents were enrolled with Medicare but only 17% had private health insurance. English was the primary language spoken at home for most (92.4%), with 28% rating their English reading ability as poor-fair.

**Table 2:** Socio-demographic characteristics, subjective health status, healthcare seeking behaviour, cancer experience and screening history of study participants

**Variable**	**n**	**%^**
**Gender (n = 93)**		
Male	22	23.7
Female	71	76.3
**Age in years (n = 90)**		
35-44	24	25.8
45-54	43	46.2
55-64	13	14.0
65+	10	10.7
**Marital status (n = 91)**		
Married or de-facto	48	52.7
Separated/divorced/widowed	12	13.1
Single	26	28.6
Unwilling to say	5	5.5
**Highest education level (n = 90)**		
Senior high school or higher (12 years or more)	40	44.4
Some high school (8-11 years)	25	27.8
Primary school or less (7 years or less)	21	23.3
Don’t remember	4	4.4
**Employment status (n = 90)**		
Employed full-time or part-time	34	37.8
Unemployed/unable to work/other	32	35.6
Home duties	19	21.1
Unwilling to say	5	5.5
**Annual household income (n = 90)**		
Less than $20,000	42	45.2
$20,000-40,000	11	11.8
$40,000-60,000	13	14.0
Over $60,000	9	9.7
Unwilling to say	15	16.2
**Has any dependants (n = 88)**		
No	40	45.5
Yes	48	54.5
**Enrolled with Medicare (n = 90)**		
No/don’t know	3	3.3
Yes	87	96.7
**Private health insurance (n = 90)**		
No/don’t know	74	82.2
Yes	16	17.8
**Self-rated English reading ability (n = 91)**		
Poor-fair	25	27.5
Good-very good	42	46.2
Excellent	24	26.4
**Self-rated current health status (n = 90)**		
Poor-fair	43	47.8
Good-very good	46	51.1
Excellent	1	1.11
**Has a regular GP (n = 88)**		
No/don’t know	13	14.8
Yes	75	85.2
**Frequency of doctor visits (n = 91)**		
Couple of times a year or more	71	78.0
Once a year	5	5.1
Less than once a year	15	16.5
**Last time saw a doctor (n = 90)**		
Less than one month ago	47	52.2
Between one month and one year ago	36	40.0
More than one year ago/can’t remember	7	7.8
**Participated in any cancer screening in past 2 years (n = 89)**		
No/don’t know	44	49.4
Yes	45	50.6
**Had a colonoscopy in the past (n = 91)**		
No/don’t know	77	84.6
Yes	14	15.4
**Ever done FOBT/bowel cancer screening test? (n = 90)**		
No/don’t know	85	94.4
Yes	5	5.6
**Knows someone in family with cancer (n = 91)**		
No/don’t know	39	42.9
Yes	52	57.1

FOBT-faecal occult blood test; GP- general practitioner. ^Percentage calculated from valid responses indicated in parentheses adjacent to survey item.

### Health-care seeking behaviour, cancer experience and screening history

Over 80% had a regular GP and had seen a doctor within the past 12 months (Table 2). Half (50%) reported having participated in any cancer screening in the past 2 years, and 15% had undergone a colonoscopy. Only 5.6% (5/90) of respondents reported ever having done a FOBT/bowel screening test.

### Awareness and knowledge of bowel cancer and FOBT bowel cancer screening

#### General awareness

The majority (78%; 72/92) of respondents reported having heard of bowel cancer, although of those, only 15% (11/72) could give a correct description of any aspect of bowel cancer (for example, ‘cancer of the bowel or digestive system’). Less than a third were aware of what polyps were (29%, 25/86) and only 19 of the 25 (76%) who said they knew what a polyp was, were able to describe it correctly. A small proportion (14%, 13/90) of respondents reported knowing what an FOBT/bowel cancer screening test was, and even after being given a detailed explanation and description of the FOBT screening test,^1^ only 30% (27/91) said that they had heard of this test (data not shown).

Those who had heard of bowel cancer before were 6.6 times more likely (*X*^2^[1] = 7.1, p = 0.008) to have seen or heard media advertising related to bowel cancer screening, and those who had heard of the FOBT/screening test were 13 times more likely (OR = 13.5, p < 0.0001) to have been exposed to such media advertising (data not shown).

#### Knowledge of bowel cancer risk factors and signs and symptoms

Responses to individual knowledge items including identification of risk factors and signs and symptoms are detailed in Table 3. The risk factor identified least frequently was having a close relative with bowel cancer, with only 56% (52/93) of respondents recognizing this as a risk, whereas 70% (65/93) were aware of excessive alcohol consumption as a risk factor. Most (87%, 81/93) recognized bleeding from the bowel as a symptom of bowel cancer, whilst more vague symptoms were less likely to be identified. Importantly, only 57% (52/92) were aware that bowel cancer could be asymptomatic.

**Table 3:** Respondent’s knowledge of risk factors and signs and symptoms of bowel cancer

**Knowledge Item (n)**	**% Yes or Agree^**
***Risk Factors***	
Being overweight (91)	82.4
Both men and women are at risk of bowel cancer (93)	80.6
Smoking (91)	80.2
Having other types of bowel disease (92)	77.2
Diet can contribute to bowel cancer (93)	76.3
Low levels of physical activity (92)	75.0
History of polyps (93)	74.2
Risk increases with age (92)	72.8
Drinking too much alcohol (93)	69.9
Both Aboriginal and non-Aboriginal people are at similar risk (93)	69.0
Risk increases if a close relative has bowel cancer (93)	55.9
***Signs and Symptoms***	
Bleeding from the bowels (93)	87.1
Change in bowel habits such as diarrhoea/constipation (92)	76.1
Loss of weight for no reason (91)	76.0
Persistent abdominal pain/cramps (93)	75.3
Unexplained tiredness or weakness (91)	71.4
Loss of appetite (92)	67.4
Bowel cancer can be present without any symptoms (92)	56.5

FOBT- faecal occult blood test.

^Percentage calculated from valid responses.

### Factors associated with bowel cancer knowledge

Composite knowledge scores ranged from 0-18 and the mean knowledge score was 13.3 (median = 15, SD = 4.3, n = 93). Knowledge was not related to demographic variables such as age, education or income. Significant relationships existed between knowledge and having participated in screening in the past or having undergone a colonoscopy, frequency of doctor visits, and family experience of cancer. Exposure to media advertising bordered on having a significant association with knowledge of bowel cancer (p = 0.051). In addition, knowledge was also related to perceived self-efficacy and perceived susceptibility (see Table 4).

**Table 4:** Variation in knowledge score according to socio-demographic characteristics and selected attitudinal variables

	**Bowel cancer knowledge score % across knowledge tertile^ (n)**			
**Variable**	**0-11 (Low)**	**12-15 (Medium)**	**16-18 (High)**	**Significance**
**Gender (n = 93)**				
Male	31.8 (7)	13.6 (3)	54.5 (12)	X^2^ = 4.5
Female	28.2 (20)	36.6 (26)	35.2 (25)	*p* = 0.109
**Age (n = 90)**				
Up to 44 years	29.2 (7)	37.5 (9)	33.3 (8)	X^2^ = 1.1
45+	28.8 (19)	27.3 (18)	43.9 (29)	*p* = 0.601
**Marital status (n = 86)**				
Married/de-facto	22.9 (11)	41.7 (20)	35.4 (17)	X^2^ = 5.8
Widowed/divorced/ separated/single	39.5 (15)	18.4 (7)	42.1 (16)	*p* = 0.054
**Education (n = 90)**				
7 years or less (primary school or less)	40.0 (10)	32.0 (8)	28.0 (7)	X^2^ = 3.32
More than 7 years (some high school or more)	23.1 (15)	30.8 (20)	46.2 (30)	*p* = 0.190
**Annual Income (n = 75)**				
Less than $20,000	28.6 (12)	35.7 (15)	35.7 (15)	X^**2**^ = 5.5
$20,000 or more	9.1 (3)	33.3 (11)	57.6 (19)	*p* = 0.064
**Self-rated English reading ability (n = 91)**				
Poor or fair	44.0 (11)	28.0 (7)	28.0 (7)	**X**^**2**^ = 4.3
Good, very good or excellent	22.7 (15)	31.8 (21)	45.5 (30)	*p* = 0.116
**Participated in any screening in last 2 years (n = 89)**				
No/don’t know	43.2 (19)	34.1 (15)	22.7 (10)	X^2^ = 12.97
Yes	15.6 (7)	26.7 (12)	57.8 (26)	***p*** = 0.002*
**Last time you saw a doctor (n = 89)**				
In the past year	23.2 (19)	31.7 (26)	45.1 (37)	X^2^ = 12.79
A year ago or more	85.7 (6)	14.3 (1)	0 (0)	***p*** = 0.002*
**Frequency of doctor visits (n = 91)**				
Once a year or less	42.3 (11)	25.0 (7)	5.4 (2)	X^2^ = 12.34
Couple of times a year or more	21.1 (15)	29.6 (21)	49.3 (35)	***p*** = 0.002*
**Ever had a colonoscopy? (n = 91)**				
No/don’t know	33.8 (26)	32.5 (25)	33.8 (26)	X^2^ = 11.05
Yes	0 (0)	21.4 (3)	78.6 (11)	***p*** = 0.004*
**Seen or heard advertising about bowel cancer (n = 91)**				
No/don’t know	35.6 (21)	32.2 (19)	32.2 (19)	X^2^ = 5.96
Yes	15.6 (5)	28.1 (9)	56.3 (18)	*p* = 0.051
**Knows someone in family with cancer (n = 91)**				
No/don’t know	46.2 (18)	30.8 (12)	23.1 (9)	X^2^ **=** 12.6
Yes	15.4 (8)	30.8 (16)	53.8 (28)	***p*** = 0.002*
**Perceived self-efficacy**				
*How confident do you feel to complete FOBT test* (n = 92)				
Not confident- somewhat confident	44.2 (19)	32.6 (14)	23.3 (10)	X^2^ =13.05
Confident-very confident	14.3 (7)	30.6 (15)	55.1 (27)	***p*** = 0.001*
**Perceived susceptibility**				
*Thinks they are at risk of bowel cancer* (n = 91)				
No/don’t know	84.6 (22)	75.9 (22)	48.6 (18)	X^2^ =10.37
Yes	15.4 (4)	24.1 (7)	51.4 (19)	***p*** = 0.006*

*Significant at 0.05.

**^**Percentage calculated from valid responses indicated in parentheses adjacent to survey item.

X^2^**-** Chi-square.

### Attitudes towards bowel cancer and screening

Perceived susceptibility to bowel cancer was low in our sample with only one-third (32%, 29/91) believing they might be at risk, and only 14% (13/92) reporting that their chance of getting bowel cancer was either high or very high (Table 5). Perceptions of severity indicated that over three quarters (77%, 69/90) felt it was a serious disease agreeing with the statements, ‘*If you had bowel cancer your whole life would change’*. Despite this, most (82%, 75/92) believed ‘*bowel cancer can be cured if found early’.* Fatalistic attitudes prevailed among a large proportion of respondents who agreed that getting bowel cancer was a death sentence (42%, 39/92 ) and that nothing can be done to prevent getting the disease (35%, 32/91). One quarter (24%, 22/91) also reported feelings of shame associated with a bowel cancer diagnosis. Only about half (53%, 49/92) rated their confidence (perceived self-efficacy) to carry out the FOBT as confident or very confident.

**Table 5:** Attitudes/perceptions towards bowel cancer and FOBT screening

**Survey Item (n)**	**%**
**Perceived susceptibility**	
*Think they are at risk of bowel cancer* (n = 91)	
No/don’t know (62)	68.0
Yes (29)	32.0
*Worried or concerned about getting bowel cancer in the future* (n = 91)	
No/don’t know (40)	44.0
Yes (51)	56.0
*Chance of getting bowel cancer in the future* (n = 92)	
Very low-low (28)	30.4
Medium (20)	21.7
High-very high (13)	14.1
Don’t know/unsure (31)	33.7
**Perceived self-efficacy**	
*How confident do you feel in your ability to do the FOBT test?* (n = 92)	
Not confident at all (13)	14.1
Not very confident (16)	17.4
Somewhat confident (14)	15.2
Confident (32)	34.8
Very confident (17)	18.5
**Fatalistic attitude**	
*Getting cancer is like being sentenced to death* (n = 92)	
Disagree (40)	43.5
Agree (39)	42.4
Don’t know/unsure (12)	13.0
*There is nothing you can do to stop yourself getting bowel cancer* (n = 91)	
Disagree (42)	46.2
Agree (32)	35.2
Don’t know/unsure (17)	18.7
**Perceived severity**	
*Do you think bowel cancer can be cured if found early?* (n = 92)	
No/don’t know (17)	18.5
Yes (75)	81.5
*If you had bowel cancer your whole life would change* (n = 90)	
Disagree (12)	13.3
Agree (69)	76.7
Don’t know/unsure (9)	10.0
*The thought of bowel cancer scares you* (n = 91)	
Disagree (7)	7.7
Agree (76)	83.5
Don’t know/unsure (8)	8.8
**Shame**	
*You would feel ashamed if you found out you had bowel cancer* (n = 91)	
Disagree (54)	59.3
Agree (22)	24.2
Don’t know/unsure (15)	16.5

FOBT- faecal occult blood test.

^Percentage calculated from valid responses indicated in parentheses adjacent to survey item.

Agree represents % of respondents who answered strongly agree or agree to survey item.

Disagree represents % of respondents who answered strongly disagree or disagree to survey item.

Generally, respondents agreed with most statements of benefits associated with FOBT screening (data not shown). The items respondents most frequently agreed with in terms of barriers to screening were being afraid to find out something is wrong (44%, 41/93) or finding out they have cancer (39%, 35/91) and not understanding how to do the test (36%, 34/93). Not having symptoms (31%, 29/91) or privacy to do the test (26%, 24/91) were also reported as constraints by about a quarter of respondents. Not having an appropriate place to store stool samples was an issue for substantial numbers of participants (40%, 37/93), as was feeling well and not having symptoms (31%, 28/93), having to mail the kit back (29%, 27/93) and not having a family history of bowel cancer (26%, 24/93).

### Intention to screen with FOBT

Overall, 63% (58/92) of respondents reported they would consider doing the FOBT screening test in the next 6-12 months if they received it (Table 6). Interestingly, a larger proportion said they would do the test if their doctor recommended it (84%; 77/92). Those intending to complete an FOBT test were significantly more likely to do a test if a doctor recommended it, while those who said they would not consider doing a test were more likely not to do a test even if a doctor recommended it (*X*^2^[1], n = 92)= 30.6, *p* < 0.0001), suggesting that those refusing to consider FOBT screening remained unlikely to do so despite a doctor’s recommendation (data not shown).

**Table 6:** Socio-demographic, knowledge and attitudes associated with intention to screen for bowel cancer with FOBT

**Survey item**	**Total (n)**	**% Yes (n)**^**a**^	**OR (95% CI)**	***p*-value**
**Gender (n = 92)**				
Male	21	61.9 (13)	1	0.902
Female	71	63.4 (45)	1.07 (0.39-2.91)	
**Age in years^**^+^ (n = 90)				
Up to 44	24	54.2 (13)	1	0.277
45+	66	66.7 (44)	1.69 (0.65-4.39)	
**Marital status^**^+^ (n = 86)				
Married or de-facto	48	81.3 (39)	5.96 (2.26-15.71)	<0.0001*
Widowed/divorced/single	38	42.1 (16)	1	
**Employment status (n = 91)**				
Employed	34	79.4 (27)	3.14 (1.15-8.58)	0.022*
Not employed	49	55.1 (27)	1	
**Education (n = 90)**				
7 years or less (primary school or less)	25	48.0 (12)	1	0.043*
8 or more years (some high school or more)	65	70.8 (46)	2.62 (1.02-6.78)	
**Annual Income (n = 75)**				
Less than $20,000	42	57.1 (24)	1	0.048*
$20,000 or more	33	78.8 (26)	2.79 (0.99-7.84)	
**Self-rated English reading ability (n = 90)**				
Poor or fair	24	54.2 (13)	1	0.277
Good, very good or excellent	66	66.7 (44)	1.69 (0.65-4.39)	
**Last time you saw a doctor^**^+^ (n = 89)				
In the past year	82	68.3 (56)	5.39 (0.98-29.6)	**0.047**^#^*
Over a year ago	7	28.6 (2)	1	
**Frequency of doctor visits (n = 91)**				
Once a year or less	20	50.0 (10)	1	0.148
Couple of times a year or more	71	67.9 (48)	2.09 (0.76-5.7)	
**Participated in any cancer screening in past 2 years^ (n = 89)**				
No/not sure	44	47.7 (21)	1	0.003*
Yes	45	77.8 (35)	3.83 (1.53-9.6)	
**Self-rated current health status (n = 90)**				
Poor-fair	43	67.4 (29)	1	0.439
Good-excellent	47	59.6 (28)	0.71 (0.3-1.69)	
**Ever had a colonoscopy^**^+^ (n = 91)				
No/don’t know	77	58.4 (45)	1	0.014*
Yes	14	92.9 (13)	9.24 (1.15-74.28)	
**Seen media advertising on bowel cancer screening**^^+^ (n = 91)				
No/don’t know	59	57.6 (34)	1	0.100
Yes	32	75.0 (24)	2.20 (0.85-5.72)	
**Knows someone in family with cancer**^^+^ (n = 90)				
No/don’t know	38	50.0 (19)	1	0.025*
Yes	52	73.1 (38)	2.71 (1.12-6.56)	
**Ever heard of bowel cancer before**^^+^ (n = 91)				
No/don’t know	20	45.0 (9)	1	0.048*
Yes	71	69.0 (49)	2.72 (0.99-7.5)	
**Ever heard of bowel cancer screening test**^^+^ (prompted) (n = 90)				
No/don’t know	63	57.1 (36)	1	0.027*
Yes	27	81.5 (22)	3.30 (1.11-9.83)	
**Aware bowel cancer/polyps can be asymptomatic** (n = 90)				
No/don’t know	40	50.0 (20)	1	0.016*
Yes	51	74.5 (38)	2.92 (1.21-7.07)	
Bowel cancer knowledge^+^				
*Bowel cancer knowledge score* (n = 92)				
0-11 (Low)	26	26.9 (7)	1	**X**^**2**^**[2]=20.3**
12-15 (Medium)	29	75.9 (22)	8.50 (2.5-28.7)	
16-18 (High)	37	78.4 (29)	9.80 (3.1-31.6)	<0.0001*
**Perceived self-efficacy^**				
*How confident do you feel to complete FOBT test* (n = 92)				
Not confident-somewhat confident	43	34.9 (15)	1	<0.0001*
Confident-very confident	49	87.8 (43)	13.40 (4.6-38.6)	
Perceived susceptibility^				
*Thinks they are at risk of bowel cancer* (n = 91)				
No/don’t know	62	56.5 (35)	1	0.035*
Yes	29	79.3 (23)	2.96 (1.06-8.3)	
**Perceived severity**				
*Do you think bowel cancer can be cured if found early*?^^+^ (n = 92)				
No/don’t know	17	35.3 (6)	1	0.009*
Yes	75	69.3 (52)	4.15 (1.37-12.57)	
*The thought of bowel cancer scares you* (n = 82)^b^				
Disagree	7	57.1 (4)	1	0.703^#^
Agree	75	64.0 (48)	1.33 (0.28-6.40)	
*If you had bowel cancer your whole life would change* (n = 80)^b^				
Disagree	11	81.8 (9)	2.72 (0.55-13.5)	0.312^#^
Agree	69	62.3 (43)	1	
**Fatalistic attitude**				
*Getting cancer is like being sentenced to death* (n = 79)				
Disagree	40	75.0 (30)	2.32 (0.89-6.03)	0.082
Agree	39	56.4 (22)	1	
**Shame**				
*You would feel ashamed if you found out you had bowel cancer* (n = 76)^b^				
Disagree	54	77.8 (42)	5.05 (1.74-14.7)	0.003*
Agree	22	40.9 (9)	1	
**Perceived benefits**				
*Doing a bowel cancer screening test can reduce chance of dying from bowel cancer* (n = 83)^b^				
Disagree	6	66.7 (4)	1	1.0^#^
Agree	77	67.5 (52)	1.040 (0.18-6.06)	

FOBT - faecal occult blood test. OR – odds ratio.

^*^Significant at p ≤ 0.05.

^#^Fisher’s Exact Test.

^^^Variables included in first step of Model 1 of multivariate logistic regression.

^+^Variables included in first step of Model 2 of multivariate logistic regression.

^a^Refers to percentage of respondents who answered yes to the screening intent item, *Would you consider doing an FOBT/bowel screening test in the next 6-12 months?*

^b^Excludes those who selected don’t know/unsure.

### Factors affecting screening intention

Table 6 summarises the relationship between intention to screen with FOBT and a number of socio-demographic, knowledge and attitudinal variables.

#### Socio-demographic factors

Those who intended to participate in screening were more likely to be married or in de-facto relationships (OR = 5.96, 95% CI = 2.3-15.7), employed, (OR = 3.14, 95% CI = 1.2-8.6) have a higher income (OR = 2.8, 95% CI = 0.99-7.8), at least 8 years of education (OR = 2.6, 95% CI = 1.0-6.8), and have seen a doctor within the past year (OR = 5.4, 95% CI = 0.98-29.6). Participation in other types of cancer screening (OR = 3.8, 95% CI = 1.5-9.6) and knowing a family member with cancer (OR = 2.7, 95% CI = 1.1-9.9) also increased the likelihood of screening uptake. Those who had previously had a colonoscopy were nine times more likely (OR = 9.2, 95% CI = 1.2-74.3) to consider screening.

#### Bowel cancer knowledge

A direct association was observed between bowel cancer knowledge and intention to screen, with the proportion intending to screen increasing as knowledge score increased. Compared to those with a low knowledge score, respondents with medium knowledge were 8.5 times (p < 0.0001) more likely to consider screening and those with high knowledge score were nearly 10 times (p < 0.0001) more likely to consider screening. Those who were aware bowel cancer can be asymptomatic, and that bowel cancer can be cured if found early, were also significantly more likely to consider screening.

#### Attitudes/perceptions

One of the most significant associations with screening intention was perceived self-efficacy or confidence in carrying out the self-screening test. Those who felt confident or very confident were over 13 times more likely to screen (OR = 13.4, 95% CI = 4.6-38.6). A greater perceived risk was also related with intent to screen (OR = 2.96, 95% CI = 1.1-8.3), as was perceived severity but only for one of three items (*Do you think bowel cancer can be cured if found early?* OR = 4.15, 95% CI = 1.4-12.6). No significant association was observed between having a fatalistic attitude or greater awareness of benefits of screening. Those who did not perceive a bowel cancer diagnosis to be shameful were five times more likely to consider taking up screening (OR = 5.05, 95% CI = 1.7-14.7).

### Multivariate analyses

The first multivariate logistic regression model which excluded bowel cancer knowledge score, identified being aged 45 or more, having greater perceived self-efficacy, and having participated in screening in the past as independent predictors of intention to screen with FOBT. In the second model, bowel cancer knowledge score and marital status remained significant independent predictors of screening intent (Table 7).

**Table 7:** **Multivariate analysis**^#^**of predictors of intent to participate in CRC screening with FOBT**

**Model 1 (excluding variable: bowel cancer knowledge score from the model) (n = 89)**		
**Predictor**	**Adjusted OR (95% CI)**	***p*-value**
Age (>44 years vs ≤44 years^^^)	4.5 (1.2-16.5)	0.026*
Participated in screening in the past (yes vs no^^^)	6.8 (2.0-23.3)	0.002*
Perceived self-efficacy (confident-very confident vs not confident^^^)	19.8 (5.5-71.8)	<0.0001*
**Model 2 (excluding the variables: perceived self-efficacy, perceived susceptibility and previous participation in cancer screening from the model) (n = 86)**		
Marital status (married vs unmarried^^^)	6.91 (2.1-22.5)	0.001*
Bowel cancer knowledge score (overall)		<0.0001*
Medium vs Low^^^	9.97 (2.4-41.3)	0.002*
High vs Low^^^	13.6 (3.4-54.0)	<0.0001*

OR- odds ratio; CI- confidence interval; FOBT- faecal occult blood test.

^#^Multivariate analysis using binary logistic regression with a backwards step-wise approach. Outcome variable refers to the item, ≤*Would you consider screening using FOBT in the next 6-12 months if a kit was given or sent to you?*’

^^^reference category.*significant at 0.05.

The following variables were excluded from multivariate analyses due to:

1. High number of missing responses: *Income*, *Shame*, *Perceived benefits*, and *Fatalistic attitude.*

2. p > 0.30 in bivariate analysis: *Gender*, *Self-rated current health status*, *Perceived severity* (*The thought of bowel cancer scares you* and *If you had bowel cancer your whole life would change)*.

3. Taken into account in the total knowledge score variable, *Aware bowel cancer/polyps can be asymptomatic*.

4. *Bowel cancer knowledge score* was excluded from Model 1 due to strong association with *perceived self-efficacy*, *perceived susceptibility* and *participated in screening in the past*.

5. *Perceived self-efficacy*, *perceived susceptibility* and *participated in screening in the past* excluded from Model 2 due to correlation with *bowel cancer knowledge score*.

6. *Self-rated English reading ability* and *Employment status* excluded due to strong association with *Education*.

7. *Frequency of doctor visits* excluded due to high correlation with *Last time saw doctor*.

8. *Education* excluded due to high correlation with *Marital status*.

9. *Marital status* excluded from Model 1 due to unstable OR and wide CI.

## Discussion

This is the first published study to examine awareness, knowledge, attitudes and behavioural intention in regard to bowel cancer screening in the Indigenous Australian population. Given significantly lower levels of CRC screening participation by this population group, it is critical to elucidate reasons behind lower uptake to ensure good evidence informs program policy makers in the design of ongoing and future health interventions.

### General awareness

This study indicates that among the population we sampled, general awareness of CRC and CRC screening was low. Most had heard about bowel cancer but were not able describe any particular detail of what it was or what polyps were, and the vast majority had not heard of bowel cancer screening or the FOBT test. The evaluation of Australia’s NBCS pilot program also showed that inadequate understanding and awareness of bowel cancer were major impediments to the participation of Aboriginal people in bowel cancer screening [35].

Having seen media advertising on bowel cancer screening was significantly associated with greater awareness and higher overall bowel cancer knowledge scores, consistent with findings reported by Schroy et al. examining the effect of media on awareness [36]. Despite this, media exposure was not an independent predictor of screening intention among our respondents, suggesting that although it has a critical role to play, it is insufficient on its own. Exposure to media promotions prior to receiving a screening kit was found to be an important trigger for participation for Indigenous people in the final evaluation of the NBCSP pilot program in 2002. This suggests a pivotal role of mass media for public education and raising the profile of bowel cancer [36] and confirms the recommendations of its use to raise awareness and ultimately facilitate screening participation in minority groups [37].

### Bowel cancer knowledge

Knowledge of specific bowel cancer risk factors was reasonable, with about two thirds or more of participants identifying the main risk factors. However, that over half of participants did not know bowel cancer can be asymptomatic, suggests this is an important concept to be emphasised in education and awareness campaigns.

Factors significantly associated with bowel cancer knowledge included having participated in cancer screening in the past, seeing a doctor more often, having a family or personal experience with cancer, having been exposed to media advertising about bowel cancer screening, greater levels of perceived self-efficacy and perceived susceptibility. Most of these factors are related to exposure to experiences that facilitate greater cancer awareness and knowledge, implying that knowledge can be gained and is not necessarily determined by education, income or age.

### Perceived susceptibility to bowel cancer

Perceived susceptibility to CRC was generally quite low, with only a third of our sample believing they were at risk despite the majority being aged over 45 years, and is in line with findings elsewhere in the literature [32]. Greater perceived risk to a disease can in some cases be associated with more positive preventive behaviour [38] and our study also showed that those with a greater perceived risk were significantly more likely to consider FOBT participation. However, perceived risk was not an independent predictor of screening intention, supporting the theory that despite being important in influencing CRC screening behaviour, it requires other mediating factors to have an effect [39].

### Intention to screen using FOBT

Intention to screen for bowel cancer was relatively high among our respondents with almost two-thirds reporting they would undertake FOBT screening in the future if they received a kit. This is much higher than the 17% participation rate observed from the most recent report of Australia’s NBCSP [9], but equivalent to that found in a study of rural Australians and low-income, ethnically diverse groups in the US [31,40]. An even greater proportion of respondents said they would do the FOBT test if their doctor recommended it - a response which supports research that shows endorsement of screening by a primary care practitioner can facilitate participation [32,41,42]. These results suggest that doctors and possibly other trusted health providers have an important role to play in encouraging screening adherence. It also supports findings from the NBCSP evaluation which demonstrated that Indigenous people needed extra support and encouragement to participate in CRC screening [35].

Bivariate analyses indicated that intention to take up FOBT screening was significantly higher among those who were married or in de-facto relationships, employed, had at least eight years of education, higher income, and a history of undergoing cancer screening or a colonoscopy. Existing research also shows that lower CRC screening uptake is consistently observed among those who are less educated and from lower income groups, and those from non-English speaking backgrounds [20,43-45]. In contrast with the literature, English reading ability was not related to screening intention in our study [46], however, this measure was self-reported. Moreover, respondents with greater overall knowledge and awareness of bowel cancer and the screening test, greater perceived self-efficacy and perceived susceptibility to the disease were also more likely to consider screening, as were those who personally knew someone with cancer.

On multivariate analysis, significant predictors of screening intention were, being aged 45 years or more, having greater levels of perceived self-efficacy or confidence in carrying out the FOBT, and past participation in cancer screening. Past screening participation was also demonstrated to be an independent correlate of CRC screening uptake in several studies [29,47], and although our research does not directly measure participation, intention to participate can provide some indication of behaviour.

One of the strongest associations and independent predictors of screening intention in our sample was perceived self-efficacy. This is highly pertinent to the Australian program as bowel cancer screening is entirely based upon a self-screening kit delivered by post, which in urban areas is to participant’s homes, although mail is not delivered to homes in some rural and remote areas. Self-efficacy has not been explored extensively in the literature as countries such as the US and UK promote alternative methods for CRC screening such as colonoscopy or sigmoidoscopy alongside FOBT. Feufel et al [48] have shown that improved instructions accompanying self-screening FOBT tests can help facilitate appropriate of test completion. This is particularly relevant for Indigenous Australians who had a higher proportion of incorrectly completed FOBT tests compared to the general population [20].

In the second logistic regression model, bowel cancer knowledge and marital status remained independent predictors of intention to screen. Knowledge and awareness can be important determinants of screening behaviour, as a greater understanding of a disease, its risk factors and methods of prevention affects an individual’s decision to participate in screening. Lower knowledge levels are associated with a lower perceived risk and poorer CRC screening adherence rates in studies with ethnic minorities [32]. An Australian study also found that those born overseas had poorer bowel cancer knowledge scores compared to those born in Australia, with knowledge a predictor of screening intent [42]. Our research finds a strong relationship between bowel cancer knowledge score and intention to screen for CRC, with screening intention increasing with each increase in knowledge tertile. This relationship was retained in multivariable analyses and is line with other research demonstrating knowledge to be a significant predictor of bowel cancer screening uptake [29,47,49]. Enhancing knowledge can also lead to more positive attitudes towards the disease and reduce negative perceptions, which could in turn impact positively on screening uptake [49]. Health education and promotion should therefore focus on improving overall knowledge related to bowel cancer and screening as a means to facilitate screening compliance.

### Screening intent and fatalistic attitudes

Previous studies have reported associations between screening intent and fatalistic attitude towards bowel cancer [50,51], but this was not evident in our study despite the large proportion of respondents possessing a fatalistic attitude. Shame was strongly associated with intention to screen in bivariate analyses but was not an independent predictor of screening intent. Concerns about shame and embarrassment differed from those observed in studies of Latinos [32], yet corroborated findings from other ethnic minority groups [52-54]. This shows the need for culturally targeted education and screening promotion campaigns to address the differing beliefs and views towards bowel cancer screening that exist between different cultural groups in order to deliver appropriate messages that are effective.

### Perceived benefits to screening

Most participants agreed with the benefits of CRC screening, although this did not appear to influence screening intent among our sample, and is unlikely to be a successful focal point in prevention or education campaigns. Similar findings have been observed in a study of African Americans [28]. Why this is the case needs to be explored in greater depth, but it may be related to not understanding personal risk or that perceived barriers have a larger impact on screening decisions.

### Barriers to FOBT screening

Fear of finding out something is wrong or finding out they had cancer were major barriers to FOBT screening for over a third of our sample. This is similar to research undertaken with Italian migrants in Australia that found fear of cancer and finding out they have cancer were major barriers to screening [53]. In the present study, additional barriers related to the design of the screening program and test method including the postal distribution, storage of samples, and lack of privacy in which to do the test. The absence of symptoms and not having a family history of bowel cancer were also reasons that respondents felt discouraged them from screening. Again, the concept of self-efficacy arose with ‘not knowing what to do’ being reported by over a third of respondents as a major reason they would not complete the screening test. These barriers cannot be ignored and most are heavily impacted by the way the screening program is designed and delivered to the Australian population. Research into test preferences for Indigenous people is an area that is under-investigated and is warranted if screening rates for bowel cancer are to be increased [55,56].

### Limitations

Several limitations to this study must be noted. As our sampling strategy was non-random, the results of this study cannot be considered representative of all Indigenous Australians. Participants were recruited primarily through respected community members working in health settings and are therefore likely to be more health connected, proactive in their health behaviour, better informed about health issues and have greater exposure to prevention messages. Furthermore, participants lived in urban and regional centres so results may not reflect the views of those living in very remote regions and living traditional/nomadic lifestyles.

The over-representation of females limited our ability to find differences in knowledge or intention to screen across gender. The nature of gender relations in Aboriginal culture may have influenced our ability to recruit male participants as well as the fact that all researchers involved in data collection and recruitment were female. The higher proportion of females and persons unemployed or not working in this study occurred because these groups are more likely to have the time to take part.

Our main outcome measure was largely hypothetical, asking participants on their future ‘intention’ to take up CRC screening. Such questions may not translate or predict real life behaviours, so results need to be interpreted with caution. Nevertheless, considering undertaking a preventive behaviour is a first step towards behaviour modification and therefore remains important.

Additionally, knowledge items from the survey were based on recognition rather than recall, limiting how far this information can be extrapolated. We felt that this approach would elicit greater responses than recall on its own, and it is more commonly used in studies with ethnic and minority groups [32,33].

Finally, our small sample size was a major limitation on the extent of analysis that could be conducted, and is partially responsible for the wide confidence intervals observed particularly in the multivariate analysis. Despite these limitations, our results have generated important information on Aboriginal views of bowel cancer and bowel cancer screening in an otherwise unexplored area of Indigenous health.

## Conclusions

Critical to developing interventions for addressing the underutilisation of CRC screening among Indigenous Australians is an understanding of the factors that influence or determine screening adherence. Our study identifies key areas of focus for future health promotion and education strategies and provides policy makers an insight into specific areas that need to be addressed in regard to the way the program is currently being delivered.

Less than six percent of our sample reported having completed an FOBT test which is surprising given that the majority were in the age group targeted by the NBCSP, and is substantially lower than what has been reported from the NBCSP and lower than Indigenous participation in other countries [9]. This may be a reflection of the inappropriate design of the program which is not reaching those high risk age groups it is designed to [4]. All respondents reported being enrolled with Medicare and should have successfully received a screening kit if address details were up to date. A large proportion of our sample were between 45-54 years of age and according to a Cancer Council report [11], screening kits were being targeted to Aboriginal people from the age of 45 years. It is also possible that the short time since the NBCSP had operated in WA (beginning in 2007) and the recall of kits in late 2009 may have contributed to the low rate reported for having received a kit.

The design of national screening programs often do not take into account the social and cultural diversity of the population in which they are implemented. A recent analysis of Australia’s NBCSP described several inequities in access to screening for Indigenous and minority groups in relation to the way the program is designed and disseminated to the public [4]. The postal distribution of the FOBT and requirement for individuals to self-screen, means the decision to carry out the test at home is made without health provider consultation. Our study also showed that these are significant barriers to screening participation. Inadequate consideration is given to low literacy populations, and the finding that self-efficacy is a major determinant of intention to screen lends further support to this.

Our study also illustrates the importance of improving individual’s knowledge on bowel cancer and confidence to carry out the FOBT test as a means to facilitate CRC screening uptake with FOBT among Indigenous communities. Health education strategies need to focus on demonstrating how the test is done to increase potential participants’ confidence levels, as well as informing community members of the risk factors and signs and symptoms, emphasising the absence of symptoms that can occur with CRC. Our study also supports the role of the media in raising awareness and knowledge on bowel cancer screening and its potential as a mediator of screening uptake. Greater media promotion can assist in eliminating stigma and shame associated with talking about an issue that may be a source of embarrassment and sensitivity for many. The attention that comes with having a celebrity or well respected community member speak about cancer can also be beneficial and encourage screening uptake [57,58].

Future studies should focus on targeting a larger cohort of Aboriginal people including those living in remote and very remote regions whose views can differ significantly from urban and regional populations. The NBCSP was implemented in WA only two years prior to this study, which meant that few participants had actually received an FOBT test kit making detailed exploration of reasons for uptake or non-screening difficult. Future research when the population is more familiar with the screening program may provide greater clarity of reasons impeding or facilitating uptake of screening.

### **Notes:**

^1^ Explanation of FOBT given to respondents: ‘*An FOBT is a screening test for bowel cancer that is carried out on healthy people with no symptoms. This test can pick up tiny amounts of blood in a sample of your bowel motion that you yourself may not notice. Blood in the stools is one sign of bowel cancer. This test is posted to you in the mail so that you can do it at home by yourself by taking a small sample of two bowel motions using sticks provided in a kit. You then return this kit by mail to be tested. After it is tested you and your GP are notified by mail of your result. If your test is positive for blood then it will need further investigation and you will be referred to have a colonoscopy at the nearest hospital.*’

## Competing interests

The authors declare they have no competing interests.

## Authors' contributions

AC coordinated the study, participated in the study design, carried out the data collection and analysis, and drafted the manuscript. SCT was involved in the initial conception of the work, assisted in the conduct of the study, sourcing funding and in refinement and editing of the manuscript. Both authors read and approved the final manuscript.

## Pre-publication history

The pre-publication history for this paper can be accessed here:

http://www.biomedcentral.com/1471-2458/12/528/prepub

## References

[B1] Condon JR Barnes T Armstrong BK Selva-Nayagam S Elwood MJ Stage at diagnosis and cancer survival for Indigenous Australians in the Northern Territory Med J Aust 2005 182 6 277 280 15777142 10.5694/j.1326-5377.2005.tb06700.x

[B2] Levin B Lieberman DA McFarland B Smith RA Brooks D Andrews KS Dash C Giardiello FM Glick S Levin TR Screening and surveillance for the early detection of colorectal cancer and adenomatous polyps, 2008: a joint guideline from the American Cancer Society, the US Multi-Society Task Force on colorectal cancer, and the American College of Radiology CA Cancer J Clinician 2008 58 130 160 10.3322/CA.2007.0018 18322143

[B3] Australian Cancer Network Clinical practice guidelines for the prevention, early detection and management of colorectal cancer 2005 Australian Cancer Council and Australian Cancer Network, Sydney http://www.nhmrc.gov.au/_files_nhmrc/publications/attachments/cp106_0.pdf

[B4] Christou A Katzenellenbogen J Thompson S Australia's National Bowel Cancer Screening Program: does it work for Indigenous Australians? BMC Publ Health 2010 10 1 373 10.1186/1471-2458-10-373 PMC2915957 20579344

[B5] International Cancer Screening Network Inventory of Colorectal Cancer Screening Activities in ICSN Countries 2008 National Cancer Institute, U.S National Institute of Health, U.S http://appliedresearch.cancer.gov/icsn/colorectal/screening.html

[B6] Department of Health and Ageing Australia's Bowel Cancer Screening Pilot and Beyond. Final Evaluation Report 2005 Commonwealth of Australia, Canberra

[B7] Flitcroft KL Salkeld GP Gillespie JA Trevena LJ Irwig LM Fifteen years of bowel cancer screening policy in Australia: putting evidence into practice? Med J Aust 2010 193 37 42 20618113 10.5694/j.1326-5377.2010.tb03739.x

[B8] Australian Institute of Health and Welfare & Australian Government Department of Health and Ageing National Bowel Cancer Screening Program Monitoring Report 2007 2008 Australian Institute of Health and Welfare, Canberra Cancer series no. 40. Cat. no. 35

[B9] Australian Institute of Health and Welfare & Australian Government Department of Health and Ageing National Bowel Cancer Screening Program: Annual Monitoring Report 2009 2009 Australian Institute of Health and Welfare, Canberra

[B10] Hol L van Leerdam ME van Ballegooijen M van Vuuren AJ van Dekken H Reijerink JCIY van der Togt ACM Habbema JDF Kuipers EJ Screening for colorectal cancer: randomised trial comparing guaiac-based and immunochemical faecal occult blood testing and flexible sigmoidoscopy Gut 2010 59 62 68 10.1136/gut.2009.177089 19671542

[B11] The Cancer Council Australia National Cancer Prevention Policy 2007-09 2007 The Cancer Council Victoria, Melbourne, Victoria http://www.cancer.org.au/File/PolicyPublications/NCPP/NCPP0709-UPDATED.pdf

[B12] Barrett T Bowel cancer screening program under threat in Australia Lancet Oncol 2011 12 2 123 10.1016/S1470-2045(11)70023-7 21355127

[B13] Wenham S Russel L Why Bowel Cancer Screening is a Needed Health Care Investment 2011 Menzies Centre for Health Policy, Australian National University and University of Sydney http://www.menzieshealthpolicy.edu.au/other_tops/pdfs_pubs/Australia-needs-a-bowel-cancer-screening-program.pdf

[B14] Department of Health and Ageing Expansion of the National Bowel Cancer Screening Program 2012 Australian Government Department of Health and Ageing; 2012, Canberra Updated 14 June 2012 [ http://www.cancerscreening.gov.au/internet/screening/publishing.nsf/Content/bowel-about]

[B15] Jorgensen O Kronborg O Fenger C A randomised study of screening for colorectal cancer using faecal occult blood test. Results after 13 years and seven biennial screening rounds Gut 2002 50 29 32 10.1136/gut.50.1.29 11772963 PMC1773083

[B16] Mandel JS Church TR Bond JH Ederer F Geisser MS Mongin SJ Snover DC Schuman LM The effect of fecal occult-blood screening on the incidence of colorectal cancer N Engl J Med 2000 343 22 1603 1607 10.1056/NEJM200011303432203 11096167

[B17] Pignone MP Flitcroft KL Howard K Trevena LJ Salkeld GP John JBS Costs and cost-effectiveness of full implementation of a biennial faecal occult blood test screening program for bowel cancer in Australia Med J Aust 2011 194 4 180 185 21401458 10.5694/j.1326-5377.2011.tb03766.x PMC3136747

[B18] Mandel JS Church TR Ederer F Bond JH Colorectal cancer mortality: effectiveness of biennial screening for fecal occult blood J Natl Cancer Inst 1999 91 5 434 437 10.1093/jnci/91.5.434 10070942

[B19] Kronborg O Fenger C Olsen J Jorgensen O Sondergaard O Randomised study of screening for colorectal cancer with faecal-occult-blood test Lancet 1996 348 1467 1471 10.1016/S0140-6736(96)03430-7 8942774

[B20] Australian Institute of Health and Welfare and Australian Government Department of Health and Ageing National Bowel Cancer Screening Program: Annual Monitoring Report 2008 2008 Australian Institute of Health and Welfare, Canberra Cancer series 44 Cat no 40

[B21] Braun KL Fong M Kaanoi ME Kamaka ML Gotay CC Testing a culturally appropriate theory-based intervention to improve colorectal cancer screening among native Hawaiians Prev Med 2005 40 619 627 10.1016/j.ypmed.2004.09.005 15850857 PMC2914227

[B22] Agrawal S Bhupinderjit A Bhutani MS Boardman L Nguyen C Romero Y Srinvasan R Figueroa-Moseley C Colorectal cancer in African Americans Am J Gastroenterol 2005 100 515 523 10.1111/j.1572-0241.2005.41829.x 15743345

[B23] Kelly KM Dickinson SL DeGraffinreid CR Tatum CM Paskett ED Colorectal cancer screening in 3 racial groups Am J Heal Behav 2007 31 5 502 513 10.5993/AJHB.31.5.6 PMC4465257 17555381

[B24] Szczepura A Price C Gumber A Breast and bowel cancer screening uptake patterns over 15 years for UK South Asian ethnic minority populations, corrected for differences in socio-demographic characteristics BMC Publ Health 2008 8 1 346 10.1186/1471-2458-8-346 PMC2575216 18831751

[B25] Deutekom M Rijn AF Dekker E Blaauwgeers H Stronks K Fockens P Essink-Bot M-L Uptake of faecal occult blood test colorectal cancer screening by different ethnic groups in the Netherlands Eur J Publ Health 2009 1 3 10.1093/eurpub/ckp051 19372193

[B26] McMichael C Kirk M Manderson L Hoban E Potts H Indigenous women's perceptions of breast cancer diagnosis and treatment in Queensland Aust New Zeal J Publ Health 2000 24 515 519 10.1111/j.1467-842X.2000.tb00502.x 11109689

[B27] Janz N Becker M The health belief model: a decade later Heal Educ Q 1984 11 1 47 10.1177/109019818401100101 6392204

[B28] James AS Campbell MK Hudson MA Perceived barriers and benefits to colon cancer screening among African Americans in North Carolina: how does perception relate to screening behavior? Canc Epidemiol Biomarkers Prev 2002 11 529 534 12050093

[B29] Lawsin C DuHamel K Weiss A Rakowski W Jandorf L Colorectal cancer screening among low-income African Americans in East Harlem: a theoretical approach to understanding barriers and promoters to screening J Urban Health: Bull New York Acad Med 2006 84 1 32 44 10.1007/s11524-006-9126-6 PMC2078250 17186375

[B30] Green PM Kelly BA Colorectal cancer knowledge, perceptions, and behaviors in African Americans Cancer Nurs 2004 27 3 206 215 10.1097/00002820-200405000-00004 15238806

[B31] Emmons K Puleo E McNeill L Bennett G Chan S Syngal S Colorectal cancer screening awareness and intentions among low income, socio-demographically diverse adults under age 50 Canc Causes Contr 2008 19 1031 1041 10.1007/s10552-008-9167-0 PMC2756999 18478340

[B32] Cameron KA Francis L Wolf MS Baker DW Makoul G Investigating Hispanic/Latino perceptions about colorectal cancer screening: a community-based approach to effective message design Patient Educ Counsel 2007 68 145 152 10.1016/j.pec.2007.04.004 17517486

[B33] Balajadia RG Wenzel L Huh J Sweningson J Hubbell FA Cancer-related knowledge, attitudes, and behaviors among Chamorros on Guam Cancer Detect Prev 2008 32 Suppl 1 S4 15 18359579 10.1016/j.cdp.2007.12.002 PMC2441600

[B34] Department of Health and Ageing Department of Health and Ageing Knowledge, attitudes & practices pre- and post-intervention surveys (2002 & 2004). Bowel cancer knowledge, perceptions and screening behaviours: final report 2005 Commonwealth of Australia, Canberra

[B35] Department of Health and Ageing Australia’s Bowel Cancer Screening Pilot and Beyond. Final Evaluation Report 2005 Commonwealth of Australia, Canberra

[B36] Schroy PC Glick JT Robinson PA Lydotes MA Evans SR Emmons KM Has the surge in media attention increased public awareness about colorectal cancer and screening? J Community Health 2008 33 1 9 10.1007/s10900-007-9065-5 18080203

[B37] MacKenzie R Chapman S McGeechan K Holding S Australian television coverage of colorectal cancer Psycho-Oncology 2009 19 3 283 288 19382099 10.1002/pon.1567

[B38] Lipkus IM Lyna PR Rimer BK Colorectal cancer risk perceptions and screening intentions in a minority population JAMA 2000 92 10 492 500 PMC2608546 11105730

[B39] McQueen A Vernon SW Rothman AJ Norman GJ Myers RE Tilley BC Examining the role of perceived susceptibility on colorectal cancer screening intention and behavior Ann Behav Med 2010 40 205 217 10.1007/s12160-010-9215-3 20658212 PMC3161120

[B40] Janda M Stanton W Hughes K Del Mar C Clavarino A Aitken J Tong S Short L Leggett B Newman B Knowledge, attitude and intentions related to colorectal cancer screening using faecal occult blood tests in a rural Australian population Asia Pac J Publ Health 2003 15 1 50 56 10.1177/101053950301500109 14620498

[B41] Cole SR Young G Byrne D Guy J Morcom J Participation in screening for colorectal cancer based on a faecal occult blood test is improved by endorsement by the primary care practitioner J Med Screen 2002 9 4 147 152 10.1136/jms.9.4.147 12518003

[B42] Koo JH Arasaratnam MM Liu K Redmond DM Connor SJ Sung JJY Leong RWL Knowledge, perception and practices of colorectal cancer screening in an ethnically diverse population Canc Epidemiol 2010 34 5 604 610 10.1016/j.canep.2010.05.013 20580631

[B43] Power E Miles A von Wagner C Robb K Wardle J Uptake of colorectal cancer screening: system, provider and individual factors and strategies to improve participation Future Oncol 2009 5 9 1371 1388 10.2217/fon.09.134 19903066

[B44] Weber MF Banks E Ward R Sitas F Population characteristics related to colorectal cancer testing in New South Wales, Australia: results from the 45 and up study cohort J Med Screen 2008 15 137 142 10.1258/jms.2008.008050 18927096

[B45] Doubeni CA Laiyemo AO Reed G Field TS Fletcher RH Socioeconomic and racial patterns of colorectal cancer screening among Medicare enrollees 2000 to 2005 Canc Epidemiol Biomarkers Prev 2009 18 8 2170 2175 10.1158/1055-9965.EPI-09-0104 PMC3018698 19622721

[B46] Diaz JA Roberts MB Goldman RE Weitzen S Eaton CB Effect of language on colorectal cancer screening among Latinos and non-Latinos Cancer Epidemiol Biomarkers Prev 2008 17 8 2169 2173 10.1158/1055-9965.EPI-07-2692 18708410 PMC2568081

[B47] Ng EST Tan CH Teo DCL Seah CYE Puhua KH Knowledge and perceptions regarding colorectal cancer screening among Chinese- a community-based survey in Singapore Prev Med 2007 45 332 335 10.1016/j.ypmed.2007.06.021 17707496

[B48] Feufel MA Schneider TR Berkel HJ A field test of the effects of instruction design on colorectal cancer self-screening accuracy Heal Educ Res 2010 25 5 709 723 10.1093/her/cyq015 20304976

[B49] McCaffery K Wardle J Waller J Knowledge, attitudes and behavioural intentions in relation to the early detection of colorectal cancer in the United Kingdom Prev Med 2003 36 525 535 10.1016/S0091-7435(03)00016-1 12689797

[B50] Gorin SS Correlates of colorectal cancer screening compliance among urban Hispanics J Behav Med 2005 28 2 125 137 10.1007/s10865-005-3662-5 15957568

[B51] Espinosa de los Monteros K Gallo L The relevance of fatalism in the study of Latinas’ cancer screening behavior: a systematic review of the literature Int J Behav Med 2011 18 4) 310 318 20953916 10.1007/s12529-010-9119-4 PMC3212691

[B52] Robb KA Solarin I Power E Atkin W Wardle J Attitudes to colorectal cancer screening among ethnic minority groups in the UK BMC Publ Health 2008 8 34 10.1186/1471-2458-8-34 PMC2267180 18221519

[B53] Severino G Wilson C Turnbull D Duncan A Gregory T Attitudes towards and beliefs about colorectal cancer and screening using the faecal occult blood test within the Italian-Australian community Asian Pacific J Cancer Prev 2009 10 387 394 19640179

[B54] Paddison JS Yip MJ Exploratory study examining barriers to participation in colorectal cancer screening Aust J Rural Health 2010 18 11 15 10.1111/j.1440-1584.2009.01114.x 20136809

[B55] Hawley ST Volk RJ Krishnamurthy P Jibaya-Weiss M Vernon SW Kneuper S Preferences for colorectal cancer screening among racially/ethnically diverse primary care patients Med Care 2008 46 9(S1) S10 S16 18725820 10.1097/MLR.0b013e31817d932e

[B56] Shokar NK Carlson CA Weller SC Informed decision-making changes test preferences for colorectal cancer screening in a diverse population Ann Fam Med 2010 8 141 150 10.1370/afm.1054 20212301 PMC2834721

[B57] Larson RJ Woloshin S Schwartz LM Welch HG Celebrity endorsements of cancer screening J Natl Cancer Inst 2005 97 9 693 695 10.1093/jnci/dji117 15870440

[B58] Chapman S McLeod K Wakefield M Holding S Impact of news of celebrity illness on breast cancer screening: Kylie Minogue's breast cancer diagnosis Med J Aust 2005 83 5 247 250 16138798 10.5694/j.1326-5377.2005.tb07029.x

